# The healing response of LAMax LAAC™ left atrial appendage occluder in a canine model: the potential influence of the implantation technique on the healing response

**DOI:** 10.1186/s12872-022-02731-5

**Published:** 2022-06-27

**Authors:** Xiaoxia Wu, Dongxing Ma, Tao Wan, Yuezhi Meng, Yilong Chen, Yejia Shen, Wei Huang

**Affiliations:** 1 Department of Cardiology, The Third Medical Center, Chinese PLA(People’s Liberation Army) General Hospital, 69 Yongding Road, Hai Dian District, Beijing, 100039 China; 2 Faculty of Hepato-Pancreato-Biliary Surgery, Chinese PLA General Hospital, Beijing, China; 3 ShenZhen KYD Biomedical Technology Co. Ltd., Shenzhen, China; 4 ShenZhen Cardiovascular Engineering Laboratory of Drug and Device Development, Shenzhen, China; 5 Department of Mechanical and Aerospace Engineering, Hong Kong University of Science and Technology, Hong Kong SAR, China

**Keywords:** Left atrial appendage occluder, Healing response, Implantation principle

## Abstract

**Background:**

Device-associated thrombus are potential causes for thromboembolic events post left atrial appendage closure (LAAC), and correlated with the complete endothelialization of the device surface. Our aim was to evaluate the endothelialization of LAMax LAAC™ occluder surface and analyze the potential influence of the implantation technique on the healing response.

**Methods:**

A total of 29 healthy dogs (28.0 ± 3.7 kg) were implanted with the devices successfully after ensuring COVER signs was met (Concavity of the disc, Oversizing by 20–50%, Verifying position, Ensuring stability, Residual flow < 5 mm by transesophageal echocardiographic (TEE) examination), and sacrificed at < 24 h, 1-, 2-, 3-, and 6-months. Gross examinations were conducted to evaluate healing response.

**Results:**

The mean diameters of LAA orifice measured by angiography and TEE were 19.0 ± 2.9 mm and 16.6 ± 2.9 mm (*P* < 0.05), respectively. TEE found that the discs in 18 dogs (62.1%) were completely pulled into the LAA with concavity and in 11 dogs incompletely pulled into the LAA with suboptimally concavity, while 5 of them had residual flow. Gross examinations showed that the complete endothelialization on the device surface with concaved disc was found at 1-month after LAAC. Microscopic examinations confirmed complete healing on the device with optimal closure effect.

**Conclusions:**

The good healing response and the optimal closure effect were observed using the LAMax device in a canine model by following the COVER implantation technique.

## Background

Patients with atrial fibrillation (AF) have a 3- to fivefold elevated risk for the occurrence of stroke compared with individuals without AF [1–4]. Because the left atrial appendage (LAA), a blind pouch of the left atrium (LA), accounts for approximately 90% of cardiac thrombi in patients with nonvalvular AF, it has been suggested that LAA closure (LAAC) might be useful in the prevention of ischemic stroke [1]. In a large real-world cohort of 1025 patients with AF who were at high risk for stroke, a relative risk reduction of incidence of ischemic stroke of ~ 80% was found after LAAC compared with what was expected based on the CHA2DS2-VASc score profile of the patients [4].

Although LAAC has been recognized as an attractive alternative to lifelong oral anticoagulation (OAC), it involves the device associated with technical requirements to minimize long-term thromboembolic risks post-implantation. Particularly, device-associated thrombus (DAT) and incomplete closure (i.e., presence of peri-device leak) have been implicated as potential causes of thromboembolic events after initial technically successful LAA closure [5]. According to existing data, the mean DAT incidence for overall LAAC was reported to be 3.9%; where it was 3.4% for Watchman device (Boston Scientific Inc., USA), and 4.6% for Amplatzer Cardiac Plug (ACP) or Amulet device (St. Jude Medical Inc., USA) [6]. DAT has been found on the threaded insert of the WATCHMAN device and the proximal end screw of the ACP, and the proximal connector pin in both the WATCHMAN and ACP devices, which were the nonendothelialized portions [5, 7]. Newer-generation LAAC devices have been designed to avoid these potential risk factors [6, 8–10]. A previous study has reported that thrombus formation occurs more frequently in the first few weeks after implantation, and its incidence tends to decline with complete endothelialization of the device surface [6]. Therefore, it is reasonable to argue that in early cases, thrombus formation is probably related to delayed endothelialization [11]. In late cases, it could occur secondary to mechanical factors (e.g., uncovered lobe, significant residual flow) or systemic patient factors. In most cases, transesophageal echocardiographic (TEE) is the conventional imaging approach to diagnose the DAT.

A previous pre-clinical canine study found complete endothelial cells coverage of the device with sealing of the device and LA interface at 45 days following Watchman device placement [11]. There were differences in the conformation of LAA surrounding structures with variable healing response between Watchman and ACP devices after LAAC in the canine model [12]. The complete neo-endocardial coverage of the Watchman device was observed at 28 days, while the ACP device showed an incomplete covering on the disk surface, especially at the lower edge and end-screw hub regions [12].

In the present study, LAMax LAAC™ LAA occluder (ShenZhen KYD Biomedical Technology Co. Ltd., China) was applied, whose shape of the cover-disc is similar to that of ACP. Our objective was to evaluate the endothelialization on the atrial surface of the occluder and analyze the potential influence of the implantation technique on the healing response of occluder in a canine model. Specifically, the endothelialization endpoint was defined as the achievement of full coverage on the atrial surface of the LAAC occluder by neo-intima and good connection between the occluder and surrounding tissue.

## Methods

### Animal model preparation and TEE examination

A total of 29 healthy farm dogs (22 males, 28.0 ± 3.7 kg) were implanted with the LAMax LAAC™ LAA occluders, which were provided by the ShenZhen KYD Biomedical Technology Co. Ltd., China. In the present study, the healthy canine model was chosen since this model had been tested in other dedicated LAA occluders with success and due to the similarity to the human left atrial appendage with an angle and a constricted LAA orifice similar to the human appendage [11, 12]. All dogs were provided by the Animal Experimental Center of Beijing Pinggu District Hospital, Beijing, China, and identifiable by ear tags, and the experiments were carried out in the center, which is well equip for cardiovascular interventional experiments and animal caring. All dogs were housed in the center, and randomly divided into 5 groups and euthanized at: < 24 h (n = 6), 1- (n = 6), 2- (n = 5), 3- (n = 7), and 6-months (n = 5) after LAAC. For premedication before TEE and euthanasia, after fasting for 12 h, the animals were anesthetized with xylazine hydrochloride (0.1 mg/kg) intramuscularly. For LAAC procedure and TEE examinations, the animals were under general anesthesia with propofol intravenously. A GE Vivid E9 with XD clear ultrasound system (GE Vingmed Ultrasound AS, Norway) was used. The methods of the animal model preparation and TEE examination were described detailedly in the published study [13]. The dogs were excluded from LAA occlusion if the dextrocardia, the enlarged heart with dysfunction (double plane Simpson method, EF < 30%), and the cardiac anomalies and anatomic variation were found by X-ray and TEE examinations. The device migration, peri-device leak, DAT, and the relationship to the left superior pulmonary vein (LSPV) and mitral annulus (MA) were assessed by TEE at < 24 h, 1-, 2-, 3-, and 6-months after LAA occlusion.

### LAA occlusion, device releasing, antithrombotic therapy

**Fig. 1:**
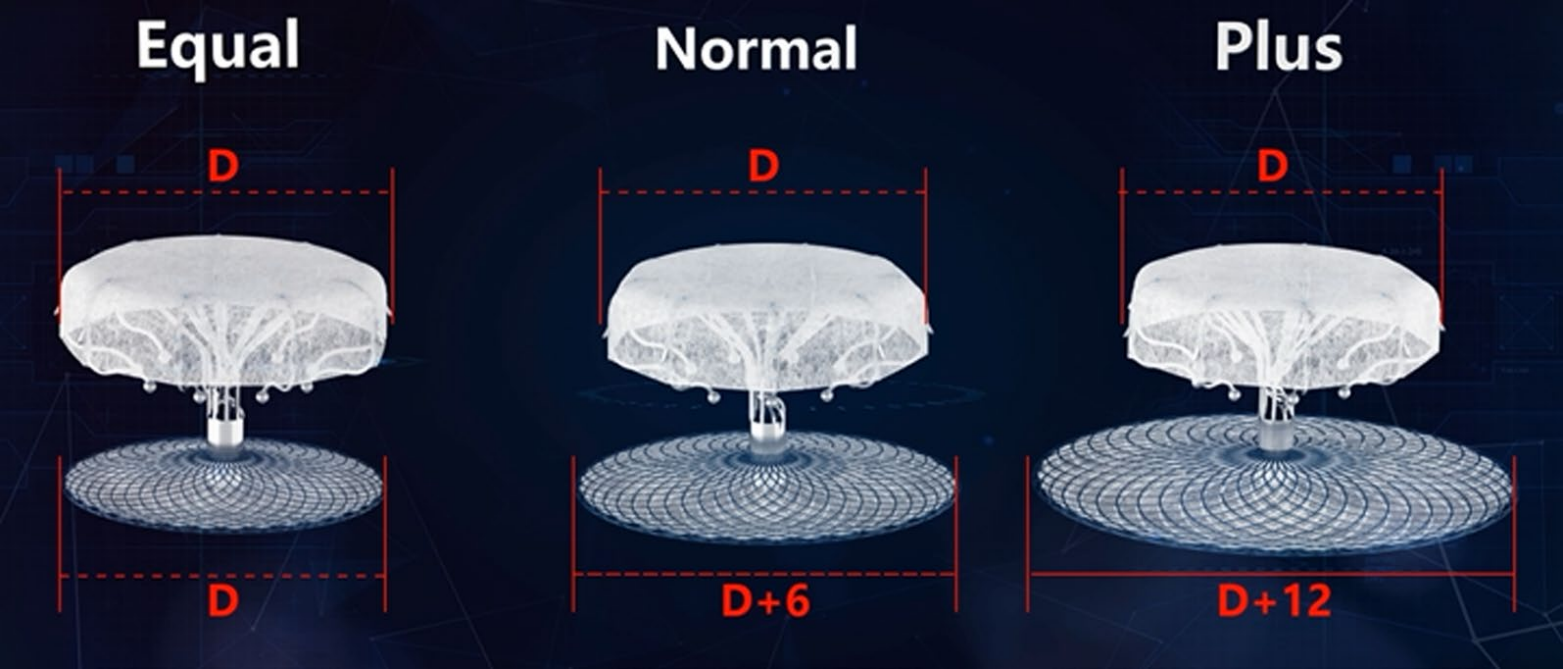
LAMax LAAC™ comprises a hook-embedded anchor and a cover-disc connected with a short central waist (10 mm), and is divided into three types: equal, normal, and plus, with the diameter differences between the cover-disc and the anchor being 0, 6 mm, and 12 mm, respectively. The cover-disc and the anchor were constructed from nitinol mesh and incorporated with Polyethylene terephthalate membrane, and both of them were specially engineered to allow for complete collapse and redeployment ex vivo. The surface of PET membrane was negatively ionized, which reduced the platelet adhesion on the atrial surface of the disc

The diameters of the LAA orifice and landing zone were measured in the right anterior oblique (RAO) cranial projection. Based on the measurements by angiography and TEE, a LAMax (Fig. 1) device was chosen. The diameter of the cover-disc was 4–8 mm larger than the measured LAA orifice and less than the distance between LSPV and MA. Prior to device release, we made sure the COVER signs was met: (1) Concavity of the cover-disc to ensure adequate sealing; (2) Oversizing, i.e., the diameter of the anchor is 20–50% larger than the measured zone; (3) Verifying the position and impingement on the surrounding structures; (4) Ensuring stability for tug test; and (5) Residual flow assessed to be < 5 mm.

A solution of Benzylpenicillin Sodium (10^5^ IU/kg/day, North China Pharmaceutical Co., Ltd, China) was intramuscularly injected within 24 h of LAAC and then daily for 7 days post-implantation. Enteric-coated aspirin 325 mg (Shijiazhuang Pharmaceutical Group Co., Ltd., China) was given 1 day before the implantation, followed by 80 mg that was given with food once per day for 4 weeks.

### Histological examination

The dogs were euthanized with an overdose of intravenous injection of pentobarbital (86 mg/kg) at < 24 h, 1-, 2-, 3-, and 6-months after being examined by TEE. The hearts were explanted by thoracotomy, and the implant was photographed in situ at necropsy. Subsequently, the LAMax and the surrounding tissue were dehydrated, infiltrated, and embedded. The LAMax was cut, polished with an EXAKT 400CS grinding system (EXAKT, Norderstedt, Germany), stained with toluidine blue, and analyzed with an Olympus BX41 microscope (Olympus Co., Lake Success, New York, USA), which was connected to the NIS-Elements imaging software (Basic Research, Version 5.20.02, Nikon Instruments Inc., Melville, New York, USA).

### Statistical analysis

Data were analyzed using SPSS version 21.0 (SPSS Inc., Chicago, IL, USA). Values were displayed as mean ± standard deviation (SD). The number of animals in groups was unequal because the animals with dextrocardia, the enlarged heart with dysfunction (double plane Simpson method, EF < 30%), and the cardiac anomalies and anatomic variation found by X-ray and TEE examinations before LAA occlusion were excluded from the present study. Paired t-test was used for pairwise comparison of homogeneous variance in normal distribution, rank sum test was used for two independent samples with uneven variance, and Pearson correlation analysis was used for correlation analysis. *P* < *0.05* was considered statistically significant.

## Results

### Measurement of LAA dimensions

The LAA orifice diameters were measured by both angiography and TEE. The mean diameters of LAA orifice measured by angiography and TEE were 19.0 ± 2.9 mm and 16.6 ± 2.9 mm (*P* < 0.05), respectively; and the correlation coefficient was 0.65. The mean length of LAA measured by angiography and TEE was 25.7 ± 5.8 mm and 23.2 ± 3.8 mm (*P* < 0.05), respectively; and the correlation coefficient was 0.59. The volume and emptying velocity of LAA measured by TEE was 3.8 ± 1.7 ml and 51.5 ± 19.8 cm/s. The mean distance from MA to LSPV measured by TEE was 26.5 ± 2.8 mm. All of the measurement data are normal distribution. When the angiographic and TEE measurements were not in agreement, the angiographic measurement was used.

### Acute procedural outcome

The LAAs in 27 dogs were successfully occluded by the first chosen device of the normal type, while in 2 cases, it was replaced by the plus type. The mean diameter of the LAMax’s cover-disc in vitro before LAAC was 25.8 ± 2.5 mm. After LAAC, the mean diameters of cover-disc measured by TEE and angiography were 23.8 ± 2.6 mm and 24.0 ± 2.8 mm (*P* > 0.05), respectively, and the correlation coefficient was 0.97, and both of them were less than that of the LAMax’s cover-disc in vitro.

### Follow-up by TEE

Scheduled TEE examinations revealed that the LAAC devices were well localized, no migration and thrombus on the cover-disc were observed. At the immediate post-LAAC, there were 5 cases of residual flow detected by color Doppler flow imaging (CDFI): 1 case with a flow width of 5 mm, and the others lower than 3 mm. Yet, the residual flow in three cases was not detected at the follow-up visit. TEE revealed that the cover-disc in 18 dogs (62.1%) was completely pulled into the LAA without residual flow and formed concavity, and in 11 dogs (37.9%) incompletely pulled into the LAA formed suboptimally concavity, while 5 of them (45.5%) had residual flow (Table 1). Additionally, TEE examinations revealed 3 cases of MA compression, which was reduced in 2 out of 3 cases at a 1-month follow-up and did not affect the movement of the posterior valve (Table 1).

**Table 1:** Case information of the cover-disc in 11 dogs without concavity revealed by TEE

Group	Case No	Device model*	LAA orifice covered by cover-disc monitored by TEE	Peri-device flow CDFI Post-LAAC (mm)				Neo-intimal coverage of LAA occluder’s atrial-surface inspected by gross examination
				< 24 h	1 m	2 m	3 m	
1 month	8	1824	Partially, gap	0	0	–	–	Partially, except area at 60° near MA
	9	2430	Partially, gap	0	0	–	–	Partially, except area at 120° near MA
	11	1824	*Partially*, Over the orifice	5	0	–	–	Partially, anchor part blocked LAA orifice, none detectable hole was found from LA to LAA
2 months	14	1824	Partially, gap	2	0	0	–	Partially, except the area at 0° near MA, none detectable hole was found from LA to LAA
3 months	18	1824	Partially, pushed MA	0	0	0	0	Fully, MA compression by cover-plate with granulation tissue
	19	2127	Partially, over the orifice	0	0	0	0	Partially, anchor part blocked small lobe, none detectable hole was found from LA to LAA
	21	1824	Partially, gap	2	2	2	2	Fully, the left edge of cover-plate inside LAA, an irregular fissure along the cover plate's edge was found and passed with an 18-gauge hypodermic needle from LA to LAA
	23	2127	Partially,Over the orifice	3	3	0	0	Partially, anchor part blocked LAA orifice, none detectable hole was found from LA to LAA
	24	1824	Partially, large gap	2	2	2	2	Fully, left edge of cover-plate inside LAA, an irregular hole was found from LA to LAA and passed with an 18-gauge hypodermic needle
6 months	25	2133^#^	Partially, pushed MA	0	0	0	0	Fully, MA compression with granulation tissue, pulmonary venous partial obstruction
	27	2430	Partially, pushed MA	0	0	0	0	Fully, MA compression with granulation tissue

^*^, the first two digits of LAMax device model are the diameter of anchor part, while the last two digits are the diameter of the cover-plate part; ^#^, LAMax plus device with a special-designed cover-plate with 12 mm larger in diameter than the anchor; LAA, left atrial appendage; MA, mitral annulus

### Anatomical examination

**Fig. 2:**
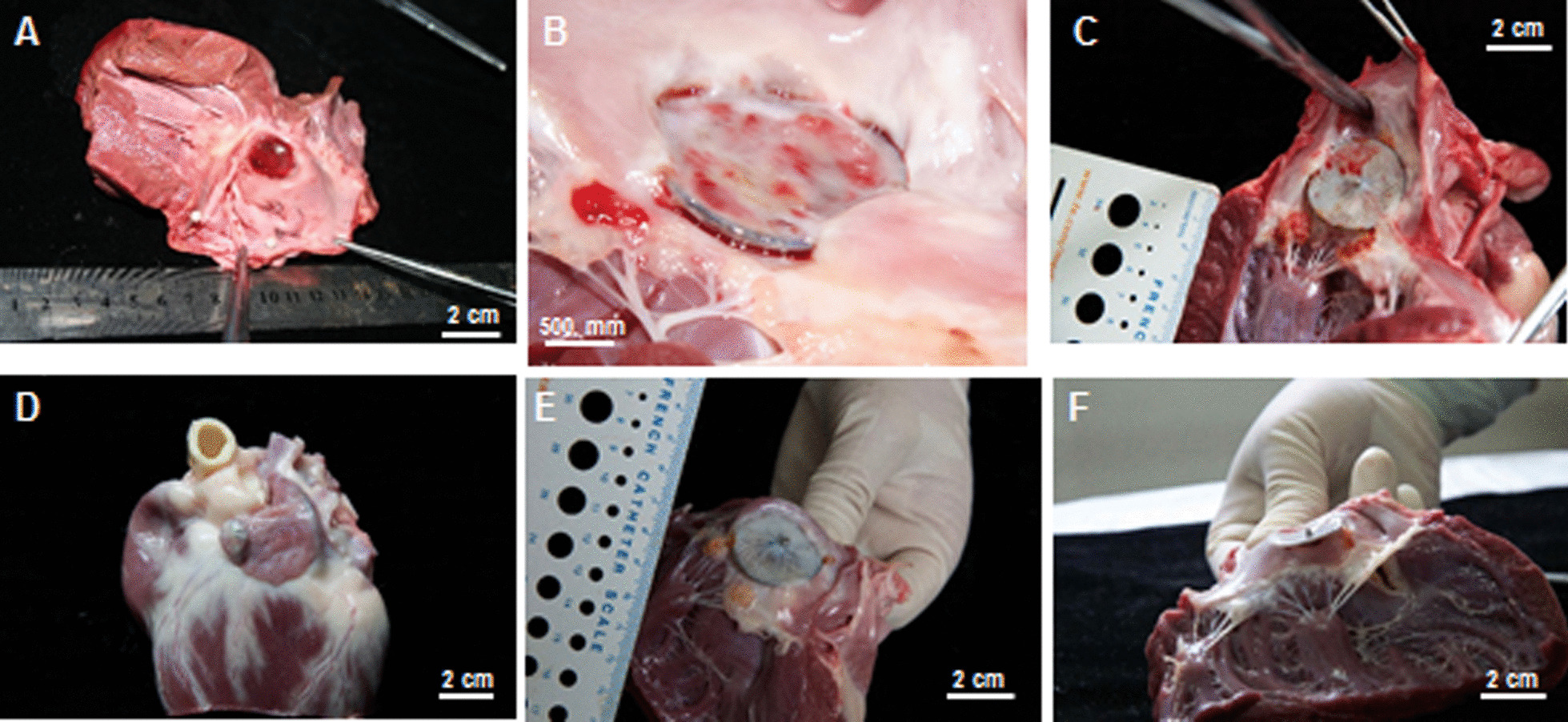
Anatomical examination. **A** The cover-disc was tightly placed on the LAA ostium, neo-intima completely covered the atrial surface of the cover-disc and the central screw hub at 1-month follow up after LAAC. **B** The cover-disc was loosely placed on the LAA ostium; although the neo-intima completed covered the atrial surface of the cover plate, the area closed to MA at 60° was not completely covered by neo-intima. **C** The neo-intima completely covered the surface of cover-disc without thrombus but did not cover the central screw hub. In the part of MA in contact with the disc, granulomas were found. **D, E** The anchor was placed in a small lobe of the bi-lobular LAA; the atrial surface of the cover-disc was completely covered by neo-intima. In the part of MA contacted with the cover-disc, granulomas were found. **F** In one dog with the residual flow at the group of 3-months after LAAC, the atrial surface of cover-disc was completely covered by neo-intima; the part of the disc at 0° connected with LA wall tightly, while the part at 180° did not connect with LA wall, and there was an irregular fissure between LA and LAA. By examining the MA side, it was found that the disc did not pull into the LAA ostium, and there was an irregular fissure between LA and LAA. In addition, an 18-gauge hypodermic needle was able to get through the hole from LA to LAA

**Fig. 3:**
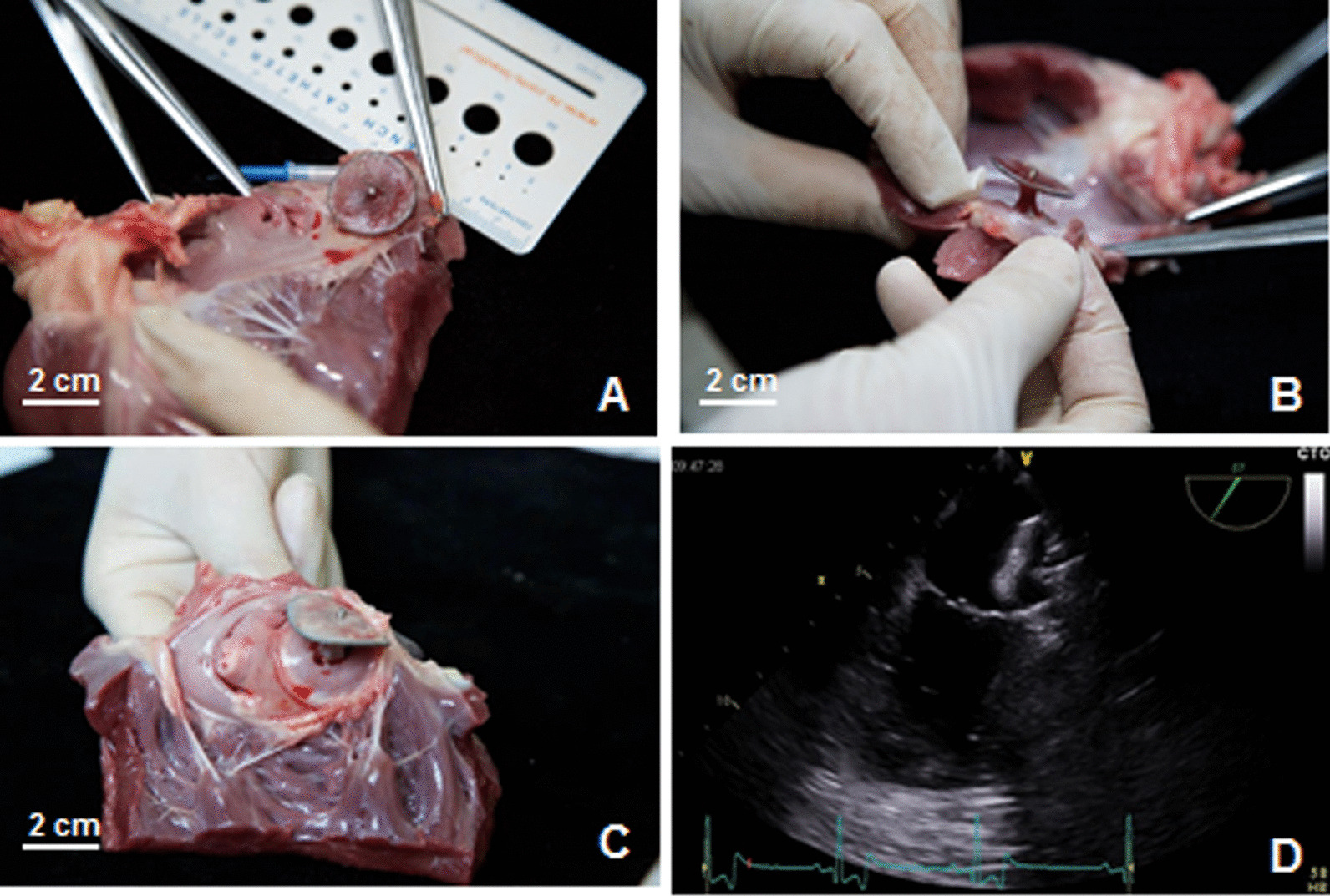
Anatomical examination of two special cases. **A, B** At 1-month follow-up, the surfaces of cover-disc and anchor were covered by a thin layer of neo-intima. The neo-intima was not found on the central screw hub but was partially found on the surface of the short central waist. **C** In the dog at 3-months follow-up, the surface of cover-disc and anchor were completely covered by neo-intima, even on the surface of the short central waist. Although the anchor was positioned at the LAA neck, the LAA was completely blocked. **D** The cover-disc was found over the LAA ostium with a certain distance, and the anchor was positioned at the LAA neck by TEE

**Fig. 4:**
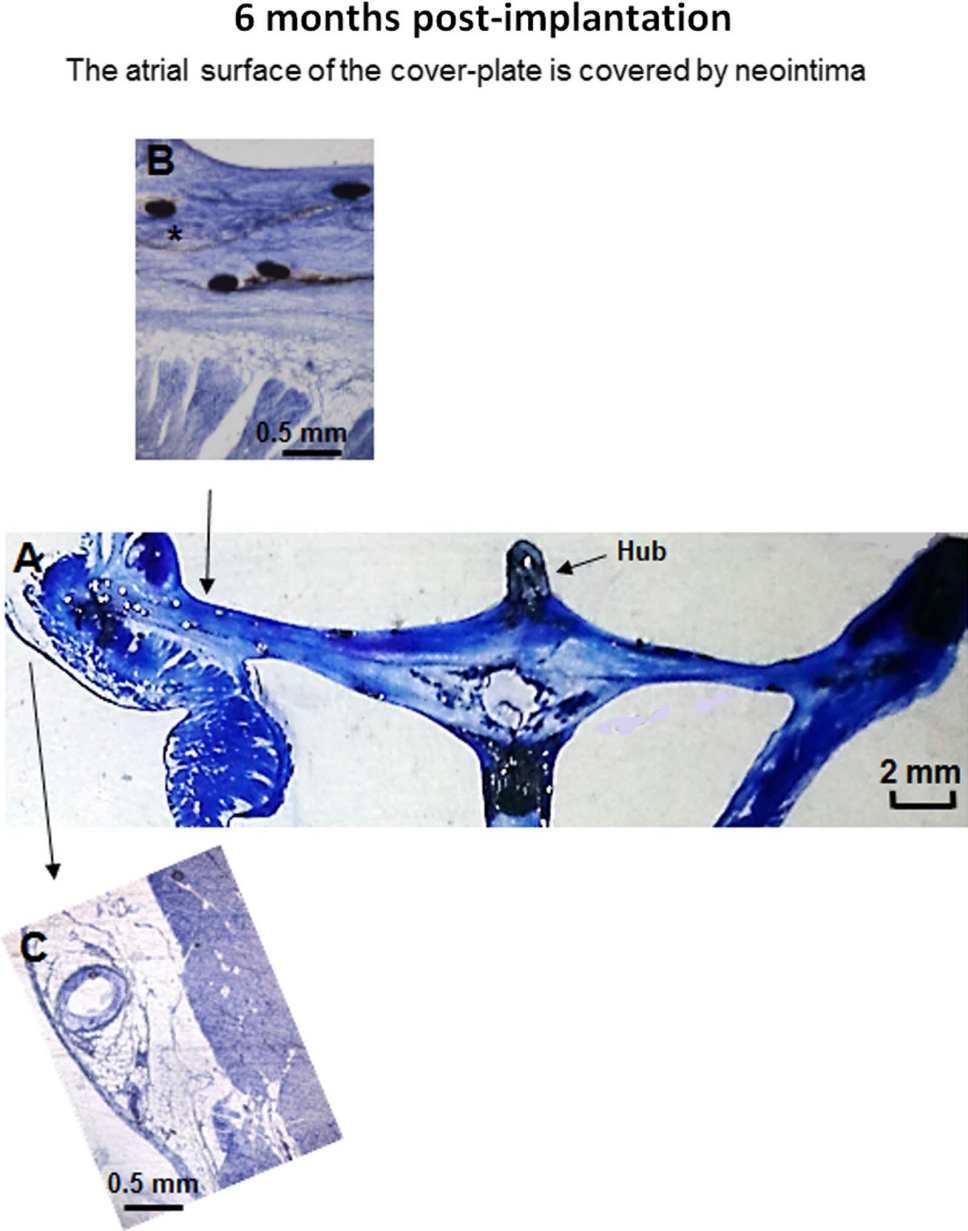
Histological examination. **A** Sagittal section through the center of the implanted device and the left atrial appendage in a dog at 6 months post-implantation (scale bar: 2 mm). **B** A close-up microscopic view showed the neointimal coverage over the atrial surface of the device, “*” indicates the PET membrane (scale bar: 0.5 mm). The tight sealing of the cover-plate with the LAA wall is shown (scale bar: 0.5 mm). **C** A close-up microscopic view showed the LAA wall without inflammation. Toluidine Blue O stain (1%)

Anatomical examinations showed that all devices were placed within LAA, the LAA ostia were well occluded. In the dogs euthanized at 1-, 2-, 3-, and 6-months after follow-up, the left atrial surface of the discs was covered by a glistening white pannus layer (Figs. 2, 3 and 4), and no thrombus was found. No visible infarcts were detected in the major organs.

### Healing response of cover-discs

The healing was faster in the dogs whose cover-disc were completely pulled into the LAA, and the neo-intima was well covered on the atrial surface of the disc and connected with surrounding tissues; therefore, the LAA was completely blocked by LAMax device. As shown in Fig. 2A, both the atrial surface and central screw hub of cover-disc was well covered by neo-intima at 1-month follow up. Yet, the healing was delayed in dogs whose cover-disc were incompletely pulled into the LAA with suboptimally concavity (Fig. 2B).

If a cover-disc which was 8 mm larger than the measured LAA orifice was incompletely pulled into the LAA, the surrounding structures would have been affected by the disc. The granulomas were found at the part of MA that was in contact with the cover-disc in one dog, and the flow of LSPV was partially blocked, but the mitral valve movement was not affected (Fig. 2C). In this case, the anchor was placed in a small lobe of the bi-lobular LAA, and the cover-disc was not pulled into the LAA, the part of MA that was in contact with the disc also found the granulomas (Fig. 2D, E).

The residual flow detected by TEE was found related to the cover-disc, which was incompletely pulled into LAA. As shown in Fig. 2F, with reference to the residual flow, the surface of the cover-disc was completely covered by neo-intima. However, the part at 180° did not connect with LA wall, and there was an irregular fissure.

The completed coverage of endothelialization on the central screw hub of cover-disc was found in 2 cases in the groups undergoing follow up at 1-month, 2 at 2-months, 2 at 3-months, and 2 at 6-months after LAAC.

### Healing response of anchor part

TEE revealed that the cover-disc was not attached to the LAA ostium in 2 out of 29 dogs with a certain distance, and the anchor was localized at the LAA neck. A thin layer of neo-intima was found on the surface of the cover-disc and anchor; and partially on the short central waist in one dog after 1-month of LAAC (Fig. 3A, B). After 3-month of LAAC, the surface of the cover-disc and the anchor were completely covered by neo-intima, and the short central waist was completely covered by neo-intima (Fig. 3C, D). Both of them had the immediate residual flow after LAAC, but disappeared because of the endothelialization of the anchor.

### LAA cavity closure

In all cases, the anchors were well localized in the LAA, and the claws of anchors were not pierced through the LAA wall. The short central waist was not broken in any case. Since the distance between the cover-disc and the anchor was short, the walls of LAA in the dogs without residual flow after 2-months of LAAC could not be separated. However, the LAA cavity of the dogs with the residual flow was still present since flow communication disturbed and delayed tissue healing.

### Histological examination

At 1-, 2-, 3-, or 6 months follow-up, the atrial surfaces in all cover-plates of the LAMax device were covered by neo-intimal layers. The histological sections showed that a moderate granulomatous inflammation was found near the woven material, the neo-intima spread into the LA wall surface, and across the device-left atrial interfaces to completely seal the LAA openings, which were covered by a thin layer of endothelial cells (Fig. 4). Endocardium, which consisted of smooth muscle cells in a proteoglycan-collagenous matrix, was found near metal and fabric (Fig. 4). The claws of the anchoring parts were well opposed to the LAA walls, with no evidence of tissue necrosis. Retention hooks were seen embedded in the LAA walls. No infarcts were observed in the major organs.

## Discussion

The present study used gross and histological examinations after LAAC in a canine model to evaluate the shape, the position, and the surrounding tissues of LAAC device. Due to its good compatibility with surrounding tissues, the coverage of neo-intima gradually developed on the atrial surface of cover-disc. A good healing response was observed in the dogs treated following the COVER implantation signs.

LAA has a complex geometric structure with an oval-shaped orifice and is placed in close proximity to LSPV, MA, and the left circumflex coronary arteries [12]. The present study found significant differences in LAA orifice diameter measured by TEE and angiography, which might be due to the oval shape of LAA orifice, the angles of TEE and angiography examinations [14]. LAAC devices are usually tested in canine models before clinical application. Nevertheless, the anatomical difference between the canine and human includes a shorter distance of the LAA ostium to MA in canine versus humans [11, 12]. In this study, the distance between MA and LSPV was larger than the diameter of the LAMax’s cover-disc in vitro. At the same time, the diameter of the cover-disc detected by TEE or angiography in vivo was less than that of the device in vitro. Therefore, the suitable LAAC device used in humans should have less influence on MA and LSPV than that observed in dogs.

LAMax and ACP devices are combined cap-on and plug-in devices. Previous studies have found that the lower edges of ACP disk extend beyond the MA and its upper edges, and the disk appears to be in loose contact with the LA wall. Additionally, histology examination showed that only a small portion of the disk surface was covered by neo-intima without significant coverage of the inferior disk edge and end-screw hub [12]. In this study, the COVER implantation signs was proposed, where “C” indicates the concavity of the cover-disc which completely pulled into the LAA, which directly affects the healing response of the atrial surface of the disc. The variability in organized neo-endocardial coverage over the devices possibly occurs due to placement within the LAA, which is consistent with a comparative study on the healing response between canines and humans [11].

The present results showed that the healing was faster in the dogs with concaved cover-disc; the endothelialization on the device’s atrial surface and central screw hub was completely achieved at 1-month after LAAC, and the concaved cover-disc did not affect MA, mitral valve movement, and LSPV flow. On the other side, the healing in dogs with suboptimally concaved disc was different, which might be related to the dimension and shape of cover-disc, whether the disc was pulled into LAA ostium and the healing time after LAAC. Particularly, in one case in this study, the cover-disc was not completely pulled into LAA ostium, and both TEE and gross examinations found the residual flow and communication between LAA and LA in the 3rd month after LAAC. Nevertheless, the atrial surface of the disc was completely covered by neo-intima. Additionally, the cover-disc which was 8 mm larger than the LAA orifice was used in the early stage and incompletely pulled into the LAA, the granulation tissue could be formed by repeated friction, and the healing could be affected and become difficult (Fig. 2C). Therefore, it is better to avoid using the too larger diameter of the device or replace it with a suitable dimension if necessary and try to pull the cover-disc into LAA ostium and achieve concavity, which promotes healing furthermore and causes less damage to the surrounding tissues. In order to ensure the concavity of the cover-disc, during the procedure, it was gently tried to pull the cover-disc into the LAA until confirming the concavity by the angiography and TEE examinations. In addition, in the two special cases where the disc did not attach to the LAA ostium (Fig. 3) occurred at the beginning of the present study because the operator lacked experience in using LAMax device. With the passing of time after LAAC, the complete coverage of neo-intima was found on the cover-disc and anchor, even on the short waist, which might be related to the design, in which both the cover-disc and the anchor of LAMax are covered with flow blocking membrane, thus forming a second defense in the process of endothelialization.

In the present study, a gross examination found that the atrial surface of the cover-disc was covered by neo-intima in the early stage, where a thick layer of neo-intima could be found in the 1st month after LAAC. Previous studies have shown similar healing responses between animals and humans; nonetheless, animal healing was faster [11, 12]. DAT with the Watchman device is almost invariably associated with the center of the device in the region exposed to the metal hub [7, 15]. According to existing study, at 200 days and 852 days after LAAC performed with the WATCHMAN device in the human heart specimen, the surface of the device was fully covered by a thick layer of neo-intima and well connected with surrounding tissues; but, the central screw was exposed without endothelialization [11]. In this study, at the 6th month after LAAC, the central screw hubs in 3 out of 5 dogs were not fully covered by neo-intima; and the reason for different response of the central screw hub remains unknown. Therefore, it is necessary to redesign the central screw hub to avoid DAT. For the prevention of thrombus, the well-known studies of the PROTECT AF and the PREVAIL suggested taking warfarin and aspirin for 45 days after LAAC, and then changing to clopidogrel and aspirin (dual antiplatelets, DAPT) therapy until 6 months. Yet, in most studies with ACP or Amulet that included patients with contraindications to OAC, the post-implantation protocol consisted of DAPT for 1–3 months followed by aspirin for ≥ 5 months [16]. Since foreign materials exposed to blood can lead to DAT, it is necessary to use continuously antithrombotic agents until the device is completely endothelialized. The present study shows that the time of complete endothelialization of the central screw hub in dogs was uncertain. The healing response in humans tends to be slower than in dogs; thus, the actual time may be longer. The ways to distinguish these patients, whether to extend the follow-up time of TEE examination and strengthen anticoagulation and/or antiplatelet therapy, need to be further addressed by the evidence-based study.

The LAA cavity was completely closed in the cases without residual flow, while in the cases with the residual flow, the LAA cavity was incompletely closed. Therefore, the residual flow should be avoided. Based on its full retrieval and repositioning capabilities, the operator can adjust the LAMax device to avoid the residual flow. Since the end portion of the WATCHMAN device expands the LAA cavity, it is difficult to complete the closure of the LAA cavity; therefore, its blockage effect can only be achieved after the full coverage of neo-intima of the device surface. This is the most important difference between WATCHMAN and LAMax devices. In our previous study, it was shown that contrast-enhanced TEE (cTEE) was superior to color Doppler flow imaging (CDFI) with respect to the blood flow communication between LA and LAA, and as a tool to supervise the closure of the LAA cavity [13].

### Study limitations

This is a single canine study without control groups, and the number of animals in groups was unequal because some dogs were excluded from the study if the dextrocardia, the enlarged heart with dysfunction, and the cardiac anomalies and anatomic variation were found. Since only 29 adult dogs were used in this study, the results and conclusion may not be applicable to a wider population. No control group was used since none commercial LAAC occluder was available in China when the present study was started in early 2014. According to the literature reports, different animal numbers were used to evaluate the feasibility and safety of LAAC occluder, and at least 3 animals were used at each time point [11, 12]. In both canine and human, the LAA has been described as narrowly tubular in shape, and lies within the pericardium, next to the superior lateral aspect of the main pulmonary artery, and superior to the left ventricular free wall. Since LAA occlusion devices usually tested in canine before clinical application [11, 12], the present study used healthy adult dogs as the animal model.

## Conclusion

The good healing response and the optimal closure effect were observed using the LAMax device in a canine model by following the COVER implantation technique.

## Data Availability

The datasets generated and/or analyzed during the current study are not publicly available due to privacy or ethical restrictions, but are available from the corresponding author on reasonable request. The study data are available in the Department of Cardiology, the Third Medical Center of Chinese PLA (People’s Liberation Army) General Hospital, please contact the corresponding author (Dr. Dongxing Ma, Email: madongxing2004@126.com).

## Abbreviations

ACP: Amplatzer cardiac plug
AF: Atrial fibrillation
CDFI: Color Doppler flow imaging
COVER: Concavity of the disc, oversizing by 20–50%, verifying position, ensuring stability, residual flow < 5 mm by transesophageal echocardiographic examination
cTEE: Contrast-enhanced TEE
DAPT: Dual antiplatelets
DAT: Device-associated thrombus
LA: Left atrium
LAA: Left atrial appendage
LAAC: Left atrial appendage closure
LSPV: Left superior pulmonary vein
MA: Mitral annulus
OAC: Oral anticoagulation
RAO: Right anterior oblique
SD: Standard deviation
TEE: Transesophageal echocardiographic
